# Noradrenergic Slow Vasomotion: The Hidden Fluid Pump Linking Sleep, Brain Clearance, and Dementia Pathogenesis

**DOI:** 10.3390/ijms262311444

**Published:** 2025-11-26

**Authors:** Marius Gabriel Dabija, Catalina-Ioana Tataru, Adrian Vasile Dumitru, Octavian Munteanu, Mugurel Petrinel Radoi, Alexandru Vlad Ciurea, Ioan-Andrei Petrescu

**Affiliations:** 1 Puls Med Association, 051885 Bucharest, Romania; mariusdabija.md@gmail.com (M.G.D.);; 2 Faculty of Medicine, University of Medicine and Pharmacy “Grigore T. Popa”, 700115 Iasi, Romania; 3 Clinical Department of Ophthalmology, “Carol Davila” University of Medicine and Pharmacy, 020021 Bucharest, Romania; 4 Department of Ophthalmology, Clinical Hospital for Ophthalmological Emergencies, 010464 Bucharest, Romania; 5 Department of Pathology, Faculty of Medicine, “Carol Davila” University of Medicine and Pharmacy, 030167 Bucharest, Romania; 6 Department of Anatomy, “Carol Davila” University of Medicine and Pharmacy, 050474 Bucharest, Romania; 7 Department of Neurosurgery, “Carol Davila” University of Medicine and Pharmacy, 050474 Bucharest, Romania; 8 Department of Vascular Neurosurgery, National Institute of Neurology and Neurovascular Diseases, 077160 Bucharest, Romania; 9 Medical Section, Romanian Academy, 010071 Bucharest, Romania; 10 Neurosurgery Department, Sanador Clinical Hospital, 010991 Bucharest, Romania

**Keywords:** noradrenergic vasomotion, locus coeruleus, perivascular clearance, aquaporin-4 polarity, glymphatic system, neurodegenerative disease, neuroinflammation, cerebrospinal fluid dynamics, neuromodulation therapy, brain–immune interface

## Abstract

Brain function is reliant upon maintaining a constant internal environment; however, the methods employed to maintain this environment have historically been viewed as largely passive in nature, relying on diffusion and vascular pulsations to create the conditions necessary for continued brain activity. This review seeks to provide an overview of current data suggesting that brain clearance is in fact an active process that is dependent upon both the current regulatory state of the brain and the presence of noradrenergic slow vasomotion, which is generated by rhythmic output from the locus coeruleus (LC). The LC-generated output has been found to influence the degree of contraction exhibited by pericytes, the geometric shape of astrocytic end-feet, and vascular tone, ultimately impacting the rate of exchange between cerebrospinal fluid (CSF), interstitial fluid (ISF), and the blood–brain barrier through aquaporin-4 (AQP4) channels. These LC-generated rhythmic changes are thought to provide the mechanical forces necessary for sustaining the metabolic clearance of waste products within the parenchyma. This review seeks to synthesize several recent studies which indicate that LC-generated vasomotion correlates with both the structure and progression of sleep states, neuronal oscillation patterns, and metabolic states, and that dysfunction of this LC-generated rhythm may contribute to pathological features associated with Alzheimer’s disease, Parkinson’s disease, and small-vessel disease. Understanding the mechanisms of clearance within the brain as a physiologically tunable system will allow researchers to view brain clearance as an adaptive neuro-modulatory function rather than merely as a passive event. Therefore, the focus of this review is on identifying the potential applications of advancements in the field of physiological imaging, molecular biomarkers, and neuro-modulatory or vascular-based therapies for early detection and therapeutic manipulation of clearance processes. Understanding these mechanisms will potentially lead to enhanced cognitive resilience and immune regulation, and promote healthy brain aging.

## 1. Introduction—Noradrenergic Vasomotion and the Hidden Dynamics of Brain Clearance

Although the brain has a very high metabolic demand that generates many chemical waste products, its ability to remove these waste products is limited by certain physical characteristics. The central nervous system (CNS) is located inside the skull, which creates a physical barrier, the blood–brain barrier (BBB), between the CNS and the rest of the body’s bloodstream. The CNS does not have the normal lymphatic network to remove waste fluids that other parts of the body use.

There was an older model that described how the brain removed interstitial solutes (substances in the spaces around cells), called the “classical model”. This model described three main components: (1) the production of cerebrospinal fluid (CSF) in the choroid plexes located in the lateral, third, and fourth ventricles; (2) the movement of CSF through the ventricular system and the spinal canal; and (3) the drainage of CSF into the veins through arachnoid granulations near the superior sagittal sinus. Pressure waves generated by the heart and lungs were thought to help in the exchange of interstitial fluid (ISF) and CSF. While the classical model was simple, it could not account for the speed of solute removal, the regional organization of clearance, or the dynamic changes in clearance rates that occur during sleep, aging, and neurodegenerative disease. As there is now a greater appreciation of the role of the buildup of metabolic waste products in the development of neurodegenerative disorders like Alzheimer’s and Parkinson’s disease, it is becoming clear that the study of the mechanisms of brain clearance will be a key area of research in modern neuroscience [1,2]. A major conceptual advancement in our understanding of brain clearance occurred with the identification of the glymphatic system. According to this model, CSF flows into the brain through periarterial channels, exchanges with ISF, and leaves the brain through perivenous routes, carrying proteins, including amyloid-β, tau, and α-synuclein. The glymphatic system requires the presence of astrocytic aquaporin-4 (AQP4) channels at the perivascular end-feet of astrocytes, which provide for rapid transmembrane water flux. The glymphatic system is most active during non-rapid eye movement (NREM) sleep, particularly during slow-wave sleep, where interstitial space increases, vascular tone decreases, and noradrenergic signaling decreases. These findings changed the way we think about the exchange of CSF and ISF, from being a passive process to being an actively regulated, state-dependent process that is tightly linked to sleep physiology [3]. One of the primary unknowns regarding the glymphatic system is what drives the fluid movement. While cardiac and respiratory pulsations contribute to the transport of CSF, they cannot explain either the magnitude or the timing of glymphatic flow. Recent studies have identified the rhythmic activity of noradrenergic neurons in the locus coeruleus (LC) as the source of slow vasomotion—the periodic changes in the diameter of blood vessels—that coordinate vascular tone, perivascular flow, and waste clearance during NREM sleep [4]. Since diffusion alone is insufficient to provide for long-distance transport of substances and since AQP4 channels do not produce force, a perivascular pump mechanism has been suggested as the means by which bulk fluid movement occurs [5].

Increasing amounts of evidence now exist that connect neuromodulation, vascular physiology, and sleep architecture. The missing link between LC activity and the oscillation of blood pressure caused by the heart during NREM sleep appears to be slow noradrenergic vasomotion—a low frequency (<0.1 Hz) oscillation in the diameter of blood vessels caused by the bursting of LC neurons [6]. During NREM sleep, the LC switches from tonic firing to bursting, causing oscillations in the concentration of extracellular noradrenaline, which then causes oscillations in the contraction of alpha-1 adrenergic receptors on vascular smooth muscle cells (VSMCs). The cyclic activation of these receptors leads to alternating increases and decreases in the concentration of intracellular calcium, leading to oscillating contractions and dilations of the blood vessel [7]. The resulting slow vasomotion generates synchronized oscillations in arteriole diameter that generate pressure differentials on the order of tens of seconds—faster than cardiac pulses, yet strong enough to cause convective CSF entry into the parenchyma [8].

In contrast to the rapid dissipation of high frequency pressure waves, slow vasomotion produces large amplitude, sustained changes in vascular tone that generate stable, directionally consistent pressure gradients. These pressure gradients travel through the compliant perivascular spaces, allowing for the convective transport of substances over a large distance [9]. Computational studies have also demonstrated that even relatively small-amplitude, low-frequency oscillations in vascular diameter can generate substantial flow velocities in combination with the hydraulics of the perivascular channels and the viscoelastic properties of the vascular wall [10]. Slow vasomotion likely works cooperatively with high frequency oscillations from cardiac and respiratory forces. High frequency oscillations provide for rapid local fluctuations, while slow vasomotion provides a sustained component that enhances total flow efficiency [11].

Experimental studies have provided a great deal of support for this mechanism. Optical activation of LC neurons at low frequency enhances vasomotion and improves the penetration of tracers into the brain parenchyma, while chemo-genetic or pharmacological inhibition of noradrenergic activity impairs vasomotion and clearance [12,13]. Dynamic contrast MRI and two-photon imaging have shown that the influx of substances into the perivascular space occurs in concert with vasomotion, and that changes in vascular diameter precede the movement of fluid [14]. Pharmacologic studies have also shown that many sedatives and hypnotics, although they promote deeper sleep and preserve high frequency pulsations, inhibit LC activity, decrease slow vasomotion, and decrease clearance [15]. Thus, it is not simply the occurrence of sleep that allows for clearance, but rather the rhythmic patterns of neuromodulation that occur during sleep.

The findings in this area of research have far-reaching implications. Neurodegenerative disorders, such as depression and anxiety, that result in dysfunctional LC activity and poor quality sleep may secondarily experience impaired metabolic clearance, which may contribute to the development of neurodegenerative disease [16]. Thus, sleep should be considered not just as a state that allows for clearance, but as a physiological program that coordinates neuromodulation, vascular resistance, and glial function to optimize fluid movement. The LC has traditionally been viewed as a center for promoting wakefulness and attention, and is now recognized as a homeostatic oscillator that controls vascular tone to allow for the removal of metabolic waste products [7,17]. Cerebral vessels do not merely serve as passive conduits, but instead act as dynamic mechanical actuators that convert neural signals into hydraulic energy. Astrocytes, in addition to their roles in neurotransmitter uptake and ion buffering, regulate hydraulic resistance in the perivascular space. Together, these structures form a state-dependent neuro-modulatory–vascular pump that integrates neural rhythms, perfusion dynamics, and metabolic homeostasis [18]. These findings have significant therapeutic implications. If impaired clearance is due to decreased noradrenergic vasomotion, therapies that increase the effectiveness of this perivascular pump may represent a new method for preventing or treating neurodegenerative disease. For example, agents that enhance the low frequency oscillations of noradrenaline in the LC or increase the sensitivity of α_1_-adrenergic receptors in VSMCs may improve the dynamics of vasomotion and perfusion [19]. Many sedatives and anesthetics, however, decrease LC activity and thus may decrease the capacity for clearance—a consideration for clinical practice.

This review aims to synthesize existing evidence related to the generation of noradrenergically induced slow vasomotion, discuss the mechanistic basis for this process, discuss the relationship between this process and neurodegenerative disease, and discuss potential therapeutic strategies to enhance or restore this process [20,21]. Increasing amounts of research conducted across molecular, physiological, and translational areas are beginning to converge on an integrated model of brain homeostasis. We hope to summarize the current state of knowledge, highlight outstanding issues, and identify the next steps in research in this area.

As stated above, the format of this review will reflect the integration of this information.

- Section 2 describes the anatomical and neurovascular organization of the noradrenergic vasomotion axis.
- Section 3 documents experimental evidence demonstrating modulation of and causality in clearance of this axis.
- Section 4 describes dysfunction of this axis in aging and disease.
- Section 5 reviews therapeutic approaches to enhance or restore the vasomotor pump.
- Section 6 outlines future directions, technological developments, and implications for translational neurobiology.

Collectively, the current findings demonstrate that brain clearance is not a passive process, but rather a dynamically regulated process influenced by the rhythmic activity of noradrenergic neurons. The LC serves not only as a generator of arousal, but as a master regulator of homeostasis, coordinating neuronal states, vascular resistances, and perivascular flow. Because each of these processes is modifiable —through sleep, adrenergic tone, or vascular compliance—dysfunction of this system may be reversible and thereby provide new opportunities for maintaining clearance and metabolic stability.

## 2. Architecture of the Noradrenergic Vasomotion Axis

### 2.1. Neural Origins and Neuro-Modulatory Dynamics

The vasomotor clearing system was developed based on a neuro-modulatory circuit that initially controlled vigilance and now has tight regulation of cerebral circulation. The LC is a small nucleus located in the pons that projects to many areas of the forebrain. It is active to varying degrees based on the state of arousal: when an organism is awake, LC neurons produce tonic activity at 1–5 Hz to maintain arousal; when the organism is asleep, particularly in deep non-rapid eye movement (NREM) sleep, LC neurons become inactive due to intrinsic membrane properties, GABAergic input from the preoptic region, glutamatergic input from the nucleus paragigantocellularis, and electrotonic coupling between LC neurons to produce infra-slow (0.01–0.1 Hz) oscillations that correspond to the slowest frequency bands of the brain during sleep (cerebral delta waves and astrocyte Ca^2+^ waves) that provide a temporal basis for state dependent transport of fluids [22,23].

LC activity influences cerebral blood flow and is primarily achieved through volume transmission. Neurotransmitters such as norepinephrine are released from varicosities of the LC and diffuse over tens of micrometers to influence the smooth muscle cells of blood vessels (VSMCs), astrocytes, pericytes and other neurons [24]. Volume transmission of neurotransmitter activity is a relatively slow process and is well suited to the mechanical characteristics of blood vessels, which tend to be most responsive to low frequency stimulation [25].

LC activity influences the activity of both alpha-1 (α_1_)- and beta (β)-adrenergic receptors on VSMCs. Activation of α_1_-receptors results in the activation of phospholipase C (PLC) and subsequent increase in intracellular calcium (Ca^2+^) resulting in contraction of VSMCs [26]. Low frequency (infralow) LC activity can result in slow vasomotion (oscillations of 2–5% of the diameter of the blood vessel at 0.01–0.1 Hz). Although the amplitude of the oscillations is relatively small, they have significant effects on the resistance of the blood vessel due to the relationship of resistance and radius of the blood vessel (resistance ∝ R^4^, where R is the radius of the blood vessel) [27]. The activity of β-receptors on VSMCs and astrocytes, however, will decrease the amplitude and possibly the phase of vasomotor cycles [28].

Astrocytes are responsible for translating neuro-modulatory signals into both structural and hydraulic changes. Astrocytes express both α_1_- and β-receptors and respond to NE with transient increases in intracellular Ca^2+^ that will alter the shape of the astrocyte and end-foot, and the distribution of AQP4 channels [29]. These alterations in the geometry and water permeability of perivascular structures will directly affect hydraulic resistance of the perivascular space. Pericytes in the capillary bed, although slower to react than astrocytes, do contract in response to NE and distribute microvascular resistance, affecting flow to different regions of the brain [30]. Together, the LC, astrocytes, and pericytes form a distributed adrenergic network that controls the vasomotor activity of blood vessels throughout the brain. The effectiveness of the adrenergic network depends on the temporal relationships between LC oscillations, cortical–thalamic oscillations, and the mechanical responses of the blood vessels and the astrocytes. During N2-N3 sleep, LC infralow activity is temporally aligned with slow waves in the cortex, spindles of the thalamus-cortex, and ripples in the hippocampus, while the blood vessels undergo rhythmic dilation, the astrocytes produce waves of Ca^2+^, and the neurons stop firing [31,32]. This hierarchical temporal structure provides a framework for the brain to maximize clearance of waste under conditions of reduced energy use and increased need for clearance of waste [33]. This system is part of larger feedback systems. Impairment of LC function due to accumulated toxins and inflammatory mediators can lead to impaired function of the adrenergic system and reduced efficiency of clearance [34]. Reduced efficiency of clearance leads to reduced levels of LC-derived norepinephrine, which, in turn, reduces the ability of the brain to clear waste [33].

Slow traveling contraction waves are generated in the blood vessel wall by synchronized Ca^2+^ transients produced in VSMCs by periodic activation of α_1_-adrenergic receptors [35]. At the same time, astrocytes modify the geometry of perivascular spaces and the distribution of AQP4 channels, thus regulating hydraulic conductivity. The combination of the oscillations of the blood vessel and glial elements generates coherent low-frequency pressure waves that propagate along the blood vessel and facilitate convective transport of fluid from superficial blood vessels to the central regions of the brain [36].

Thus, LC activity functions not only as a trigger for vasomotor waves but also as a regulator of the sensitivity of astrocytes to adrenergic stimuli and the polarity of AQP4 channels, thus continuously adjusting the efficiency of the clearance pathway [37,38].

### 2.2. Vascular Mechanics, Perivascular Hydrodynamics, and Glial Interfaces

Blood vessels transform neuro-modulatory signals into mechanical forces that drive perivascular flow. Pial and penetrating arterioles with rich walls of VSMCs are the major effectors. Slow oscillations of the diameter of blood vessels induced by low frequency NE production drive pressure waves that propagate along the blood vessel wall [39]. These waves cause displacement of CSF into the PVS and mixing of CSF with interstitial fluid (ISF); together they create convective currents of fluid through the brain [40]. Studies modeling the blood vessels suggest that optimal frequencies for producing net convective flow are in the range of 0.01–0.1 Hz [41]. PVS’s formed around penetrating arterioles small (10–30 µm), highly compliant tubes bounded by the vascular basement membrane and the end-feet of astrocytes are ideal for converting sustained pressure waves into coherent flow [41]. The interaction between pressure waves caused by vasomotion and local mechanical impedance (i.e., the product of vessel stiffness and the extracellular matrix) can enhance the transfer of fluid from the periarterial to the perivenous regions of the brain [42].

The geometry of PVSs (i.e., branching, tapering, and interconnecting) further modulates flow patterns. There are several oscillatory drivers of flow: cardiac pulses (5–10 Hz) predominate near the proximal ends of the arteries and quickly die away with distance; respiratory rhythms (0.2–0.3 Hz) modulate the pressure inside the skull and can synchronize slower waveforms; slow vasomotion (0.01–0.1 Hz) is a low frequency, high impact driver of flow that can extend flow over great distances [11,43,44]. Depending on phase, cardiac and respiratory components can either augment or dampen the gradients of flow caused by vasomotion. The interaction between cardiac/respiratory components and vasomotion can result in a multiscale, tunable clearance system that can adapt to changing physiological needs [45]. Astrocytes play a critical role in regulating the hydrodynamic environment of the blood vessels. Astrocytic end-feet that are rich in AQP4 channel proteins link the pressure in the PVSs to flow in the interstitium. Proper polarization of AQP4 channels to the end-feet of the astrocytes lowers resistance to flow and enhances convective transport of fluid from the blood vessels to the interstitium. However, depolarization of AQP4 channels in astrocytes in disease or aging can disrupt proper alignment of the channels and increase resistance to flow [46]. Astrocytes can dynamically adjust the morphology of their end-feet to modulate the geometry of the perivascular space and flow through the space, depending on the concentration of Ca^2+^ in the cell [47]. Astrocytes can also modulate the resistance of capillary beds to flow by contracting in response to adrenergic stimulation and redistributing flux to different parts of the brain. Venules, although poorly muscularized, can also participate in the generation of flow by passively oscillating in diameter at phases related to the waveforms generated by vasomotion, facilitating the exit of ISF from the brain. Optimal coordination of arteriovenous flow is necessary for effective clearance of waste; failure to achieve coordination can significantly reduce clearance [48].

Dysfunction of this axis can be a key underlying mechanism for various disease processes. Loss of LC neurons can lead to decreased oscillatory NE output, decreased vasomotor responsiveness to NE, increased stiffness of the blood vessel wall leading to decreased amplitude of vasomotion, depolarization of AQP4 channels leading to increased resistance to flow, and disrupted sleep–wake cycles disrupting the timing of vasomotion [49]. Together, these changes can lead to impaired perivascular flow, accumulation of toxic solutes, and enhanced neuroinflammation seen in aging, neurodegenerative disorders, and traumatic brain injury [50]. In order that the functional coherence of the system should be made clear, Table 1 is designed to indicate the major components of the noradrenergic vasomotion axis and their coordinated roles in effecting the cerebrovascular and perivascular fluid dynamics. It is intended to synthesize the neural, vascular and glial layers in a coherent mechanistic explanation, showing how the rhythms of norepinephrine derived from the LC are manifested in terms of oscillatory vasomotion, convective flow and adaptive clearing of solutes.

## 3. Experimental Evidence for a Tunable Noradrenergic Fluid Pump

### 3.1. Direct Manipulation of the Noradrenergic System Demonstrates Causality

Direct perturbation of LC activity is direct proof that noradrenergic vasomotion causes perivascular transport. The optogenetic stimulation of LC neurons at sleep-like frequencies (~0.05 Hz), which are low enough to be termed “infralow”, induces simultaneous oscillations in arteriole diameter and significantly increases tracer uptake into the tissue; but tonic or high-frequency stimulation does not. This clearly demonstrates that it is the temporal pattern of neuronal activity, and not the frequency of the neuronal firing, that is causal [58,59].

Similarly, chemo-genetic activation also increases vasomotion and transport, and chemo-genetic inhibition suppresses both by >50%. Both of these effects are maintained even after removal of the sympathetic innervation of the vessels, and without an increase in systemic pressure, demonstrating that the central nervous system controls vascular tone via noradrenaline [60,61,62]. Pharmacological studies have shown that alpha-1 adrenergic receptor signaling is required for vasomotion and perivascular transport. Alpha-1 blockade abolishes vasomotion and perivascular flow even though the LC is still firing; beta-blocking has little effect [63,64].

Activation of the LC in mice with reporter genes for amyloid-beta and tau accelerate their clearance; inhibition of the LC delays clearance, with clearance being proportional to the amplitude of the vasomotion [65].

Clearance peaks during NREM slow waves, showing that the physiological state affects the coherence of the pump [66,67].

Measurements of perivascular pressure demonstrate that LC activity generates oscillatory pressure gradients of several mmHg that are phase-locked to vasomotion and will produce convective flow. Models of the biophysics of perivascular flow, that include the amplitude–frequency parameters measured in vivo, accurately model in vivo tracer kinetics. Models that assume only diffusion cannot accurately model the in vivo tracer kinetics, establishing that the LC-driven vasomotion is sufficient to explain brain-wide clearance [68].

### 3.2. Imaging, Biophysical, and Pharmacological Evidence of State-Dependent Fluid Transport

Optical imaging with two-photon microscopy shows that low-frequency (0.01–0.1 Hz) oscillations in arteriole diameter consistently precede the movement of tracer molecules along the periarteriolar space by several seconds, demonstrating the temporal precedence of vasomotion relative to the movement of tracer molecules [69]. Similar dynamic contrast MRI studies show that whole-brain CSF influx oscillate at the same periodicities and peak during NREM slow-wave activity. Blocking the alpha-1 adrenergic receptors abolishes vasomotion and reduces CSF influx; stimulating the LC or administering alpha-1 agonists restores both [70,71,72]. Sedatives–hypnotics (zolpidem and propofol) decrease LC activity and abolish infralow vasomotion, although cortical slow-waves remain intact. As a result of the abolished vasomotion, the decreased LC activity eliminates the influx of tracer molecules into the perivascular space by ~60%, and eliminates the clearance of metabolites from the interstitial space, indicating that sleep EEG is not the driving force behind the clearance of solutes from the brain, but rather the adrenergic rhythm is [73]. Re-establishing alpha-1 tone restores vasomotion and clearance of solutes from the interstitial space, without affecting sleep stages, thereby establishing that the adrenergic tone is the primary modifiable variable controlling clearance [74].

Biophysical studies of structural perturbations have demonstrated that the efficiency of the pump is dependent on the integrity of the substrate. Decreasing vascular compliance by arterial stiffening results in decreased amplitude of vasomotion and decreased influx of tracers into the perivascular space, even with preserved LC activity. Increasing hydraulic resistance by reducing AQP4 polarity or function in astrocytes reduces perivascular flow; reversing AQP4 polarity or function restores perivascular flow [75,76]. Therefore, vasomotion requires coherent coordination between LC activity, vascular compliance, and astrocyte polarity. Models of the biophysics of perivascular flow that incorporate vessel elasticity, PVS geometry, and fluid viscosity accurately model the convective velocities measured in vivo when vasomotion is modeled. Impairment of any one parameter predicts deficits in the age-related and disease-related clearance [77]. Stimulating sensory input at low frequency can entrain cortical slow-wave activity, enhance vasomotion, and increase influx of tracer molecules into the perivascular space, demonstrating that the pump can be externally entrained [78,79]. Studies using two-photon microscopy and MRI resolved different scales of resolution: microscopy revealed localized micro-meter scale oscillations in arteriole diameter; MRI resolved whole-brain convective exchange. Collectively, they showed that glymphatic transport arises from a frequency-layered system in which cardiac pulsation generates localized mixing within the micro-vessels, respiratory-rhythms modulate intracranial pressure, and low-frequency vasomotion provides the persistent convective-bias [80,81,82,83].

### 3.3. Pathological Disruption, Human Evidence, and Therapeutic Tunability

Studies of disease models indicate that disruption of vasomotion precedes pathological development. In models of Alzheimer’s disease, degeneration of the LC occurs early, accompanied by decreased vasomotor amplitude and decreased influx of tracer molecules into the perivascular space; restoring either LC activity or alpha-1 tone restores clearance of solutes and delays pathological development [84,85]. Traumatic brain injury disrupts rhythmic activity of the LC and glymphatic flow; restoration of rhythmic activity of the LC restores glymphatic flow and promotes recovery [71].

Vascular stiffening, chronic sleep loss, inflammatory responses, and metabolic stress all converge on the same phenotype: decreased amplitude of vasomotion → impaired clearance of solutes [86,87,88]. Restoration of any one of these components (LC output, vascular compliance, AQP4 polarity) restores clearance [89].

Although human studies are indirect, they provide strong evidence for conservation of this system. Blood-volume and flow were imaged using functional MRI to show that infra-slow rhythms in blood volume and flow occur with sleep stage; phase-contrast MRI was used to show that CSF oscillations at 0.01–0.1 Hz occur with sleep stage and peak during NREM; neuromelanin-sensitive MRI was used to show that degeneration of the LC correlates with cognitive decline and impaired dynamics of perivascular space [90,91].

This system is very plastic. Enhancement of LC activity, enhancement of alpha-1 signaling, restoration of AQP4 polarity, or improvement of vascular compliance all enhance clearance, whereas suppression of LC activity, blockade of adrenergic receptors, or stiffening of arteries impede clearance [92]. Interventions of sensory, behavioral, or electrophysiological nature that enhance slow-wave or infralow rhythmic activity can modulate vasomotion non-invasively [93].

In addition to influencing clearance of solutes, vasomotion also influences other functions, such as perivascular immune trafficking, BBB permeability, and regulation of extracellular vesicles, placing vasomotion as a key regulator of neurovascular-immune signaling [69,94,95].

A hierarchical order of evidence now exists:

- Causal level: LC manipulation is sufficient to drive perivascular flow.
- Mechanistic level: Imaging and modeling show temporal precedence, frequency dependence, and biophysical feasibility.
- Integrative level: Pharmacology and anatomy define the variables that modulate or inhibit the system [96,97,98].

**Figure 1:**
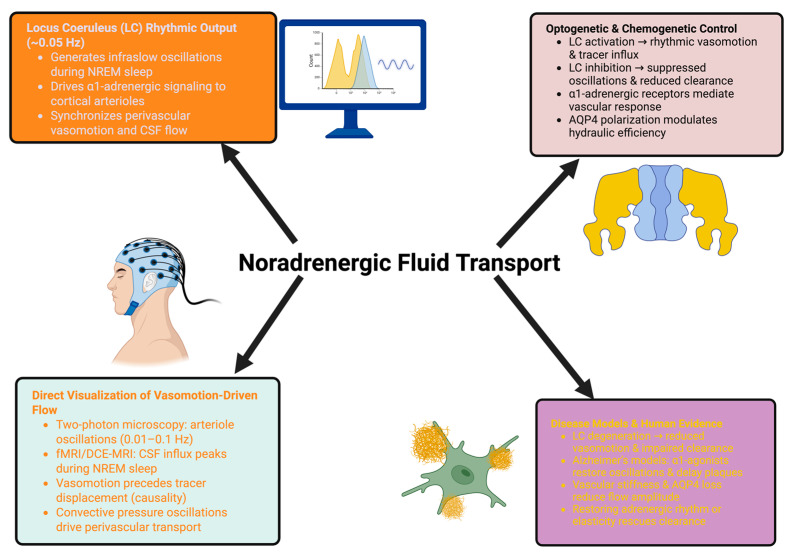
Experimental Evidence for a Tunable Noradrenergic Fluid Pump. Rhythmic LC activity at infra-slow frequencies (~0.05 Hz) during NREM sleep drives α1-adrenergic signaling in cortical arterioles, producing oscillatory vasomotion that synchronizes CSF flow. Optogenetic and chemo-genetic activation of LC neurons enhances vasomotion and solute clearance, while inhibition or α1 blockade suppresses both. Two-photon and MRI imaging show arteriole oscillations (0.01–0.1 Hz) preceding tracer displacement, confirming a causal role in perivascular transport. Disease models reveal that LC loss, vascular stiffening, or AQP4 disorganization impair clearance, whereas restoring adrenergic rhythm or vessel elasticity rescues it. Together, these findings identify LC-driven noradrenergic vasomotion as a tunable physiological pump linking neural rhythms to brain fluid dynamics.

Collectively, these studies establish that brain clearance is an actively regulated, state-dependent neurovascular process, not a passive byproduct of circulation or sleep. Failure of this system promotes neurodegeneration, and modifiability of this system presents a potentially powerful therapeutic strategy [43]. An outline of this process is suggested in Figure 1.

While preclinical data have provided compelling evidence for a role of the LC in driving vasomotor mechanisms, human validation is currently limited. The majority of evidence has been derived from optogenetic, chemo-genetic, and tracer-based studies with high levels of causal resolution, but relatively low levels of translational relevance [99,100]. Therefore, near-term progress toward the development of a predictive clinical physiology model of perivascular transport will depend upon the establishment of quantitative correlations between noradrenergic rhythmicity and measurable hemodynamic or perivascular parameters in humans. Potential methods to achieve this goal include using neuromelanin-sensitive magnetic resonance imaging (MRI) to evaluate the structural integrity of the LC, dynamic contrast-enhanced MRI to measure glymphatic flux, and diffusion-based measures of perivascular anisotropy. While indirect, these measures may provide estimates of threshold values—vascular elasticity, astrocytic permeability, oscillation coherence—whose deterioration can indicate transition from reversible to irreversible dysfunction. In order to transform the model from a conceptual framework into a predictive clinical physiology model, it will be necessary to integrate multiple variables. A significant number of translational gaps exist between rodent models of LC function and human physiology despite methodological convergence across optogenetic and chemo-genetic systems [101,102]. These differences arise due to differences in the anatomy of the LC between rodents (≈150,000 neurons) and primates (≈400,000 neurons), the length of vascular pathways, and the frequency of intrinsic oscillations (≈0.01 Hz in primates vs. 0.05–0.20 Hz in rodents), which affect the relative contributions of neural drive, vascular compliance, and hydrodynamic forces [103,104]. Additionally, many studies using optical and chemo-genetic methods use anesthesia (isoflurane, urethane) during experimentation, which depresses noradrenergic tone, increases BBB permeability, and affects vascular elasticity—factors that are known to alter vasomotor amplitude and coherence [105].

As such, human evidence remains primarily correlative and is generated by slow-fMRI, phase-contrast MRI, and neuromelanin-sensitive imaging studies demonstrating spatiotemporal vasomotor coherence, but lacking the ability to isolate the contribution of noradrenergic mechanisms [106]. Non-invasive neuromodulation methods, including focused ultrasound and transcutaneous vagal stimulation, may offer the potential to modulate LC tone indirectly, but the physiological specificity and long-term safety of these methods remain unclear [107]. As such, translational progress will be dependent upon the development of cross-species computational models that incorporate multimodal imaging (PET, fMRI, elastography) and longitudinal biomarkers (plasma NE metabolites, soluble AQP4 fragments) to determine if the quantitative relationships demonstrated in animal models are preserved in humans [108,109]. Table 2 illustrates the major experimental methodologies used to establish noradrenergic vasomotion as a primary driver of perivascular transport.

## 4. Collapse of the Vasomotor Pump in Aging and Disease

### 4.1. Degeneration of Noradrenergic Rhythms: The Erosion of Systemic Drive

LC is the first and most susceptible pillar of the vasomotor pump to degrade, often decades prior to clinical symptom development. Because LC has fewer than 50,000 neurons and a high metabolic demand due to its extensive arborization, it is particularly sensitive to mitochondrial dysfunction and impaired mitophagy as well as oxidative stress [96]. Systemic inflammatory mediators such as IL-6 and TNF-α and breakdown of the blood–brain barrier stimulate the activation of pontine microglia and exacerbate LC neurodegeneration. Selective early loss of LC neurons occurs in those neurons that project to forebrain vasculature and astro-glial territories and thus decreases the noradrenergic drive responsible for coordinating clearance [110,111]. 

With the decrease in the number of LC neurons, there is a reduction in the precision of infralow oscillations. The remaining LC neurons begin to fire abnormally due to the disruption of their hyperpolarization-activated cyclic nucleotide-gated channels, reduced T-type calcium channel currents and altered GABAergic modulation, which disrupts the hierarchical relationship between LC output, cortical slow wave and astrocytic calcium rhythm and destroys the temporal framework necessary for clearance to occur efficiently. Lower levels of alpha1-adrenergic receptor stimulation lead to decreased vasomotor gain, whereas lower beta-adrenergic receptor stimulation leads to the loss of stabilizing feedback mechanisms for oscillatory amplitude and phase [112,113,114].

Declining levels of noradrenergic stimulation propagate dysfunction throughout the glial–vascular unit. Astrocytes lose the adrenergic-evoked cytoskeletal dynamics and AQP4 clustering; pericytes demonstrate blunted contractile responses leading to reduced capillary level resistance tuning; and microglia enter a chronic state of activation leading to the release of cytokines that are toxic to LC neurons, creating a self-amplifying loop of degeneration [115]. Sleep becomes increasingly fragmented; slow-wave activity decreases; and the coherence of vasomotion during NREM sleep deteriorates. Factors in the environment impact upon this vulnerable node; chronic sleep loss impacts the inhibitory tone; hypertension and metabolic syndrome negatively impact pontine perfusion; and mild traumatic brain injury (mTBI) results in persistent microglial activation. Regardless of the condition causing it, the consistent finding is that reduced LC inflow will predict the failure of perivascular transport and neurodegenerative progression [116,117,118].

### 4.2. Vascular Stiffening and Network Remodeling: Mechanical Decoupling of the Pump

Regardless of the integrity of neuro-modulatory inputs, failure of vasomotion occurs if the vascular network cannot translate rhythmic input into mechanical movement. Chronic aging, hypertension and metabolic disorder result in the stiffening of the cerebrovascular network through fragmentation of the internal elastic lamina, loss of elastin and collagen cross-linking [119]. VSMCs transition from a contractile to a synthetic phenotype characterized by a reduction in the expression of alpha-actin and diminished Ca2+ dynamics, resulting in a decreased magnitude and fidelity of diameter oscillations. These structural changes result in reduced sensitivity to adrenergic transduction, resulting in turn in a narrow, unresponsive dynamic range. Oxidative stress, advanced glycosylation end products, and increased oxygen tension contribute to the remodeling of the extracellular matrix and calcification of the vascular wall, further decreasing vascular compliance [120]. Imaging using two-photon microscopy demonstrated that the vasomotor amplitude of penetrating arterioles in aged mice was less than 30% of the vasomotor amplitude seen in young adult mice, which correlated with the decreased perivascular flow and tracer penetration [69]. Decreased compliance results in a disruption of the frequency-layered relationship between vasomotion and cardiac pulsatility and respiratory oscillations. As vessels become stiffened, the low-frequency components of vasomotion are lost; the high-frequency components of cardiac waves become mechanically decoupled; and the multiscale pressure gradients required for convective transport are collapsed [121]. Arterio-venous phase coherence is lost, and the pressure differentials across the parenchyma flatten, leading to the necessity for the system to rely on diffusion for transport, which is inadequate for clearing amyloid-β, tau, α-synuclein, or inflammatory mediators. Remodeling of the microvasculature adds additional impedance to transport. Chronic hypertension causes arteriolar rarefaction, and increases the tortuosity, formation of micro-aneurysms and basement membrane thickening—all of which reduce the volume of the perivascular space, increase resistance and alter the distribution of microvascular load. Dropout of pericytes also disrupts the stability of capillary tone and the hydraulic architecture downstream [122,123,124].

Regardless of whether the LC output is maintained, the mechanical degradation significantly impairs the transmission of vasomotor waves [125].

Vascular stiffening is a feed-forward mechanism that promotes additional damage to the neuro-modulatory system. Stiffer vessels transmit greater pressure shocks to perivascular cells and neurons, contributing to the breakdown of the blood–brain barrier, inflammation of the brain and progressive damage to the LC [126,127].

Therefore, there is a self-reinforcing loop where the decrease in LC tone impairs mechanical transduction, and the stiffening of the vasculature continues to erode neuromodulation, ultimately leading to the degradation of the clearance pump [128,129].

### 4.3. Astro-Glial Disruption and Perivascular Interface Breakdown: The Final Bottleneck

Finally, if the astro-glial interface is damaged regardless of the preservation of neuro-modulatory input and mechanical transduction, then the exchange of CSF–ISF will be impaired. With age, and with the occurrence of disease, the morphological and functional polarity, morphology and signaling of astrocytes is destroyed, converting the perivascular interstitial gateway into a high resistance bottle-neck [130].

The primary defect lies in the depolarization of AQP4. Astrocytic reactivity, cytokines produced by microglia and destruction of the dystrophin-dystroglycan complex results in the displacement of AQP4 from the astrocytic end-feet into non-perivascular membranes, greatly increasing hydraulic resistance [131,132]. The degree of depolarization is sufficient to produce a decrease in the rate of tracer penetration of >50%, regardless of the presence of intact vasomotion [133].

Astrocytic Ca^2+^ signaling is also compromised. There is a reduction in the expression of IP_3_ receptors and diminished store-operated Ca^2+^ entry, both of which limit the extent of end-foot remodeling and the response of astrocytes to rhythmic neuro-modulatory inputs, thereby limiting the ability to dynamically adjust perivascular resistance during oscillatory flow [134,135].

Activation of microglia and the infiltration of other immune cells contribute to the worsening of the impairment. Activated microglia and infiltrating immune cells release metalloproteases and reactive oxygen species that alter the stiffness and topography of the basement membrane, redirecting flow pathways, creating zones of stasis and enhancing solute retention [136,137]. Activated perivascular macrophages enhance the inflammatory loops and distort the localization of AQP4 and glial function [138].

Impairment of glial function ripples throughout neural homeostasis. Impaired glymphatic flow reduces the delivery of ions, neuromodulators and extracellular vesicles; synaptic homeostasis is impaired; antigen clearance is impaired; and neuroinflammation becomes self-sustaining [11,139].

In virtually all neurodegenerative diseases, a similar triad is present: decreased LC drive, vascular stiffening and astrocytic depolarization shifts the system from an active convective pump to a diffusion limited architecture incapable of clearing amyloid-β, tau, α-synuclein, or inflammatory mediators [140,141,142]. Traumatic brain injury follows the same path, initially involving acute AQP4 depolarization and basement membrane remodeling followed by chronic LC dysfunction and vascular stiffening [143]. However, significant aspects of the pump can be reversed. Restoration of LC tone (chemo-genetic or pharmacologic) can restore vasomotion even in aged mice. Pharmacological or genetic intervention to target vascular stiffness (e.g., NO donors, crosslink breakers or TGF-β pathway modulators) can improve vascular compliance and mechanical transduction. Repair of dystrophin-complex defects or reprogramming of astrocytes to restore AQP4 polarity can restore perivascular water flux. Early multimodal interventions (combining neuro-modulatory, vascular and astro-glial repair) have shown synergistic effects and suggest that restoring coherence within the system may prevent or partially reverse disease progression [144,145]. Table 3 intends to outline the step-wise collapse and emerging reversibility of the pump at the neuron, vessel and glia interfaces.

As described above, vasomotor collapse is an end point for a variety of diseases; however, the initial pathophysiology differs among them. For example, degeneration of the LC occurs prior to significant amyloid deposition within the cortex in patients with Alzheimer’s disease, causing early disruption of adrenergic rhythmicity and resulting in both acute vasomotor dysfunction and disruption of aquaporin-4 function [70]. Similarly, α-synuclein pathology within the locus coeruleus and pons disrupts noradrenergic signaling, although later than that observed in Alzheimer’s disease, in patients with Parkinson’s disease [153]. In contrast, in cerebral small vessel disease, vascular injury (such as arteriolosclerosis, endothelial inflammation, or decreased vascular compliance) progressively disrupts the neural modulation of vascular response by disconnecting neural input from the mechanical transduction of the vascular response. Across all these conditions, chronic sleep fragmentation, hypertension, systemic inflammation, and metabolic syndrome are common factors that promote the progressive loss of the same axis of function: noradrenergic function, vascular stiffness, and astro-glial polarization, although each condition has its own unique temporal and spatial entry points [154].

Similarly, the loss of slow vasomotor control also contributes to a broad spectrum of neurovascular disorders through its role in disrupting cerebrovascular autoregulation [155]. Disrupted cerebrovascular autoregulation results in compromised blood flow regulation and leads to increased risk of stroke, cerebral vasculopathy, post ischemic edema and breakdown of the BBB, which result from impaired neurovascular coupling, microvascular hypoxia, and perivascular congestion without structural occlusion [156,157,158]. Therefore, vascular stiffness, endothelial dysfunction, and impaired waste clearance are united by their disruption of a single pathway of vasomotor–adrenergic function, thus establishing a direct link between neurovascular disease and neurodegenerative disease mechanisms [159].

#### Conclusion of Section 4

Therefore, the loss of noradrenergic function represents a global deterioration of the brain’s functions that spans rhythmicity of the LC, mechanical properties of the vessels, and polarity of the astrocytes. Loss of rhythmicity of the LC decreases the magnitude of the vasomotor responses; stiffening of the vessels impairs the ability of the vessels to mechanically transmit the forces generated by the vasomotor responses; and depolarization of astrocytes increases the hydraulic resistance [96]. Collectively, these coupled impairments cause the brain to transition from a coordinated, convective clearance network to a diffusion bound, static network, thereby increasing the likelihood of protein aggregation, oxidative stress, and chronic inflammation. This continuum of clearance failure defines clearance failure as dynamic and partially reversible: restoration of the rhythmicity of the LC, elastic properties of the vessels and polarity of the astrocytes may restore convective clearance and potentially modify disease progression. The transition from an ordered pulsatile clearance mechanism to a disordered diffusive clearance mechanism provides a mechanistic basis for understanding how the onset, progression, and treatment of disease are interrelated [160].

## 5. Therapeutic Reactivation of the Vasomotor Clearance System

Failure in any of the three sub-systems—LC rhythmicity, vascular compliance, or astrocytic polarity—rapidly affects the other two, and thus must be targeted by therapeutic strategies. Often, the most effective strategy will be to target the failing subsystem and, in many cases, multiple therapeutic strategies may need to be employed to recover phase coherence among all three subsystems [161,162].

In essence, these therapeutic strategies “retune” the system. Neuro-modulatory therapies adjust the gain and frequency of the system, vascular treatments restore mechanical resonance, and glial therapies restore the perivascular interface [163,164].

### 5.1. Restoring Neuro-Modulatory Rhythms: Reviving the Central Driver

The central driver is the LC. The most upstream and often the most transformative way to reactivate the pump is to restore LC rhythmicity. There are two primary adrenergic strategies for enhancing vascular responsiveness to LC output: (1) alpha-1 agonists increase vasomotor amplitude, and (2) beta-modulators increase oscillatory coherence between vascular responses to LC output. Delivery systems have been developed to deliver therapeutic agents directly to the spinal cord via the use of nanotechnology, thereby limiting side effects from systemic administration [165,166,167]. Chemo-genetic stimulation has demonstrated the ability to reinstate infra-slow oscillations and to improve clearance in both aged and diseased model systems [59]. Optogenetic entrainment of LC activity has been shown to enhance phase coherence and tracer transport when stimulated in synchrony with slow cortical waves. Although non-invasive stimulation techniques have yet to demonstrate similar efficacy to chemo-genetic and optogenetic stimulation, they represent a potential avenue for stimulation of LC activity in a clinically relevant manner [168].

Preservation and/or replacement of LC neurons represents another approach to maintain stable output. Antioxidants, mitophagy enhancers, and anti-inflammatory agents have been used to prolong the life span of LC neurons, whereas stem-cell derived noradrenergic progenitor cells have been shown to partially restore norepinephrine output in preclinical models [169,170]. Modulation of brain state has also been shown to support the maintenance of LC rhythmicity. For example, closed-loop sensory stimulation, synchronized to cortical slow waves, has been shown to strengthen endogenous oscillations, and pharmacologically inducing a deeper level of NREM sleep (e.g., GABA_B agonist, orexin antagonist) has been shown to enhance clearance conditions [171].

Gene-based therapies, including CRISPR modification of mitochondrial regulators and pacemaker channels (i.e., HCN, T-type Ca^2+^), represent an evolving new generation of therapies aimed at maintaining LC rhythmicity [172].

### 5.2. Rejuvenating Vascular Mechanics: Restoring the Hydraulic Engine

Since vessels convert LC rhythmicity into mechanical force, restoring vascular compliance is critical to the functioning of the pump. Most therapeutic strategies seek to reverse ECM-induced stiffening of vessels using lysyl oxidase inhibitors, matrix metalloproteinase (MMP) modulators, and advanced glycation end-product breakers in metabolic diseases [173,174]. Increasing the bioavailability of nitric oxide (NO) via NO donors or phosphodiesterase inhibitors increases vessel dilation and long-term elasticity and expands the dynamic range of vasomotor responses and wave propagation [175].

Furthermore, reintroducing a contractile VSMC phenotype (via modulation of myocardin, serum response factor, Notch, or PI3K-Akt pathways) has been shown to restore Ca^2+^ sensitivity and oscillatory fidelity to vessels. In addition, epigenetic interventions that reverse senescent chromatin states have been shown to further enhance contractile behavior [176]. Mechanical conditioning (e.g., sustained blood-pressure reduction or pulsed-flow conditioning) of vessels synchronizes vascular mechanics with cardiac and respiratory rhythms and restores vasomotor propagation in experimental models [177].

Advanced nanoparticle delivery systems enable selective remodeling of cerebral vessels. Elastin-stabilizing peptide or ECM-modifying enzyme nanoparticles delivered to cerebral vessels enable the localized restoration of compliance [178]. Increased elasticity of cerebral vessels reduces pathological pulse transmission, protects micro-vessels and perivascular glia, and restores the multiscale pressure gradients necessary for convective transport [179]. Clinical trials examining AGE-breakers, endothelial gene therapy, and NO-based interventions are underway and provide evidence for the translational feasibility of vascular rejuvenation. Emerging non-invasive conditioning paradigms (intermittent hypoxia, visceral pressure stimulation) are showing promise in enhancing glymphatic function [180].

### 5.3. Repairing the Astro-Glial–Perivascular Interface: Rebuilding the Clearance Gateway

The astro-glial end-foot is the last barrier to be crossed for CSF–ISF exchange; failure of this interface can prevent clearance, regardless of whether LC rhythmicity and vascular mechanics are otherwise normal. Restoration of this interface begins with repolarization of AQP4. This can be achieved by pharmacologically enhancing the expression of dystrophin and syntrophin, and by remodeling ECM anchors such as laminin and agrin [181]. Other anti-inflammatory strategies include suppressing IL-1β and TNF-α signaling, reducing astrogliosis, and stabilizing end-foot architecture. Additional therapeutic avenues exist through genetic and cell-based methods. Viral vectors that enhance AQP4 trafficking, CRISPR tools that modify AQP4 localization, and astrocyte reprogramming via STAT3, NF-κB, or Sox9 pathways facilitate restoration of perivascular polarity in models of glymphatic dysfunction [182,183].

Additionally, transplantation of glial progenitors capable of expressing polarized AQP4 is being investigated as a means to repair perivascular organization. Restoration of dynamic astrocytic Ca^2+^ signaling (via modulation of IP_3_ receptors or store-operated Ca^2+^ entry) enables real-time adjustments to perivascular resistance in response to neuro-modulatory rhythmicities [184]. Remodeling of the perivascular ECM (using enzymatic degradation of pathological collagen or hyaluronan, or through the use of biomimetic scaffolds) decreases hydraulic resistance and reopens narrow spaces. Protection of the basement membrane against damage caused by microglial NADPH oxidase or MMPs is also critical to maintaining the structural integrity of the astro-glial interface over time [185].

**Figure 2:**
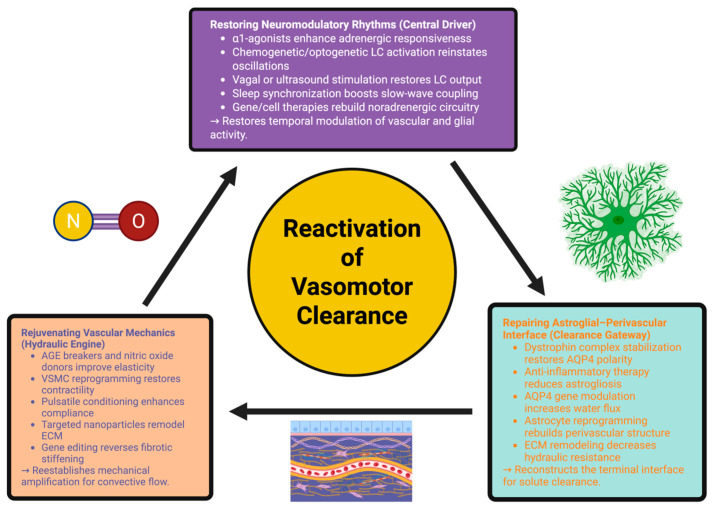
Therapeutic Reactivation of the Vasomotor Clearance System. The figure summarizes three convergent therapeutic strategies for restoring perivascular clearance. Restoring neuro-modulatory rhythms reestablishes locus coeruleus–driven oscillations; vascular rejuvenation recovers mechanical compliance for convective flow; and astro-glial repair rebuilds the perivascular interface for solute exchange. Together, these interventions reconstruct the rhythmic coupling between neural, vascular, and glial domains, reactivating the brain’s intrinsic vasomotor clearance mechanism.

In addition to supporting solute clearance, repair of the astro-glial interface supports synaptic homeostasis, neuromodulator distribution, extracellular vesicle transport, and immune surveillance. Anti-inflammatory biologics that target IL-1β and TNF-α have been shown to normalize AQP4 polarity and glymphatic flow in aged models and combination astro-glial–vascular–neuro-modulatory therapies have been shown to exhibit additive improvements in clearance [186,187]. Collectively, these results suggest a unifying therapeutic approach, in which restoration of LC rhythmicity, vascular mechanics, and astro-glial polarity converge to restore the brain’s intrinsic vasomotor flow. Figure 2 illustrates how each of these interventions contribute to an integrated recovery process.

### 5.4. Clinical Feasibility, Risks, and Translational Steps

Even though the therapeutic methods presented earlier provide a logical structure for recovering vasomotor regulation, most of these treatments are still at an early stage of translational development. Those that have the greatest potential for rapid development, based upon the existing use of clinical agents such as adrenergic receptor modulators, nitric oxide-based vasodilators, phosphodiesterase inhibitors and anti-inflammatory biologics, are those whose safety and systemic effects have been previously documented [188]. On the other hand, more complex approaches, for example, gene editing methods, nanoparticle-based targeting systems and cell replacement for the LC or astro-glial cells, need to undergo a significant amount of validation in terms of long-term safety and specificity, as well as immunological tolerance [189].

There is a unique risk for each therapeutic strategy: stimulating LC activity can negatively impact cardiovascular stability or sleep architecture; augmenting vascular compliance can disrupt autoregulation in sensitive blood vessels; and therapies targeted towards the astrocytes carry a risk of non-specific genomic or immune related side effects [175]. While non-invasive neuro-modulatory methods such as focused ultrasound and vagal nerve stimulation are encouraging, they also require further definition of safe dose levels and longer term safety information [190].

The reasonable pathway for translating these findings into humans will occur in stages. First, standardization of imaging and fluid biomarker assessments of LC health, vascular compliance and astro-glial polarity will need to be established to serve as surrogates for assessing the functionality of the pump [191]. Next, early phase studies utilizing already approved agents will be required to determine if small changes in neuro-modulatory, vascular and glial tone result in detectable increases in perivascular flow or sleep-dependent clearance. Only after safety and biological effectiveness are demonstrated should more invasive approaches such as gene editing, closed-loop neurostimulation, or cell-based repair be advanced to human trials. Ultimately, identifying which component of the clearance network is failing (i.e., neuro-modulatory, vascular, glial, or a combination) in individual patients will be critical to developing lower risk therapeutic approaches that target only the patient’s predominant failure mechanism [161].

#### Conclusion of Section 5

The noradrenergic vasomotor pump is a deep-seated, integrated, and multi-component system—and so are all the pillars of its existence susceptible to therapeutic restoration. Reviving LC rhythmicity reinstates the temporal driver of the clearance, rejuvenating vascular mechanics restores its hydraulic capability, whilst repair of the astro-glial–perivascular interface restores the conduit for solute exchange. Each component of these interventions—from gene editing through cell therapy to pharmacological modulation and conditioning of mechanical forces—shows convergent effects upon perivascular flow and solute clearance [192]. Their combined application holds the potential to modify neurodegenerative diseases from inevitable pathways of progress to modifiable ones, so radically transforming the therapeutic environment from symptomatic modulation to reinstating the brain’s own intrinsic homeostatic environment [85].

## 6. Translational Horizons and Future Directions

The Noradrenergic Vasomotion Pump offers a unifying theory of neurodegeneration in Alzheimer’s and Parkinson’s disease and represents a single systems-level failure based on loss of LC cells, stiffened blood vessels and failure of clearance. The NVP integrates findings from multiple areas, including neurodegenerative, inflammatory and body–brain interfaces, including skin–brain interfaces [141,193].

### 6.1. Clearance as a Tunable Systems-Level Function: A Paradigm Shift

Clearance is a regulated, state dependent process mediated by LC controlled vasomotion; it is not simply a passive hydraulic process. Noradrenergic rhythm, vascular biomechanical, astrocyte polarity, and perivascular geometry are part of a single tunable clearance system [194,195].

LC infra-slow oscillations (0.01–0.1 Hz,) which coordinate with cortical slow waves and astrocyte calcium oscillations, modulate vasomotion and AQP4 clustering via α1- and β-adrenergic pathways [196,197]. Studies have shown that longitudinal changes in LC degeneration, vascular stiffening and AQP4 depolarization occur prior to accumulation of amyloid, tau, and α-synuclein and therefore indicate that clearance failure is an upstream cause of neurodegenerative pathology [198,199]. These three factors define Alzheimer’s, Parkinson’s, small vessel disease, TBI, and post-infection syndromes [200].

Therefore, in order to intervene early in patients, future research needs to develop methods to quantify phase dynamics, nonlinear feedbacks and tipping points between neuro-modulatory, vascular and glial subsystems using multiscale modeling [201,202,203].

### 6.2. Tools to Measure and Modulate the Pump in Humans

Translation of the NVP requires development of tools to assess and manipulate pump function in humans [70,204].

Ultra-slow fMRI can detect sub-0.1 Hz vasomotor oscillations; 4D phase contrast MRI can resolve perivascular flow; and elastography can determine arterial compliance and therefore permit multi parametric pump mapping when used in conjunction with ASL, VASO, and neuromelanin sensitive MRI [205,206,207]. PET ligands for adrenergic receptors and astro-glial markers as well as biochemical indicators (NE metabolites and soluble AQP4) provide dynamic biomarkers [101,208].

Focused ultrasound to the LC, vagus nerve stimulation and closed loop phase locked stimulation synchronized to slow waves represent examples of neuro-modulatory interventions to activate the pump. Therapies targeted at the vascular compartment (AGE breakers, lysyl oxidase inhibitors, PDE inhibitors, and eNOS activators) aim to restore compliance and wave propagation. These are complemented by low frequency oscillatory conditioning and nanoparticle mediated ECM remodeling [209,210,211,212].

Interventions directed at the astro-glial compartment (antisense dystrophin correction, CRISPR based AQP4 relocalization, IL-1β/TNF-α anti-biologics and STAT3/Sox9 reprogramming) restore perivascular polarity and water flux [213,214]. The combined use of biomarkers will allow for early identification, stratification, and real-time monitoring of the effectiveness of therapy [215,216].

In addition, machine learning models that incorporate cognitive markers, sex-specific predictors, amnesia profiles, and LC-personality interactions will further refine individualized risk and resilience assessments [217,218,219,220].

### 6.3. Precision Neurotherapeutics and the Rebuilding of Brain Homeostasis

The NVP provides a new perspective on neurodegenerative diseases as reversible systems-level failures. Combination therapies that target the LC rhythmicity (α1/β2 agonists and NE reuptake modulators), vascular mechanics (AGE breakers, nitroxyl donors), and astro-glial polarity (AQP4 traffickers, anti-inflammatory agents) can potentially restore pump function synergistically [169,221,222].

Gene editing strategies that increase the resilience of the LC or reduce VSMC senescence can also be incorporated into neuro-modulatory devices. Closed loop systems that can sense LC activity or vasomotor oscillations and deliver adaptive stimulation represent a potential next step in maintaining clearance continuously [223]. Identifying early signs of LC, vascular or astro-glial decline allows for the implementation of prevention strategies (sleep optimization, vascular maintenance, intermittent neuro-modulatory stimulation) decades before the first cognitive symptoms occur [224,225].

Classifying neurological diseases according to failure of clearance networks (neuro-modulatory, vascular, glial or mixed) may provide a common classification for all neurodegenerative, autoimmune, post-infectious and cognitive disorders [226,227].

Restoration of perivascular flow may extend the cognitive lifespan of individuals by reducing metabolic waste, inflammation and network fragility [47].

#### Conclusion of Section 6

The LC-neuromodulator activated vasomotor pump creates a dynamic tunable homeostatic system for clearance. Neurodegenerative disease results from a simultaneous failure of LC rhythmicity, vascular compliance, and astro-glial polarity, each representing a separate therapeutic target [228]. Rapid progress is being made towards translating the NVP to clinical applications with convergence of imaging techniques, biomarkers, neuromodulation, gene editing and vascular engineering, allowing the possibility of delaying, modifying or reversing degeneration by restoring the intrinsic homeostatic rhythms of the brain [229].

## 7. Systems-Level Implications Beyond Disease

### 7.1. Cognitive Homeostasis and Network Stability: Clearance as a Prerequisite for Information Processing

The various mechanisms provided by clearance to couple brain function to the removal of metabolic waste products provide several ways in which the brain’s metabolic waste removal affects its function. First, clearance helps to maintain an appropriate concentration of ions (K^+^ and Ca^2+^) in the extracellular environment to keep neurons excited and promote coherent oscillations in the network necessary for memory formation [55]. Second, clearance clears away the metabolic byproducts produced by astrocytes, including lactate and reactive oxygen species, providing mitochondria with the resources they need to sustain the high energy levels required for long term potentiation [230]. Third, clearance limits the amount of excess neurotransmitters and inflammatory mediators that accumulate in the synaptic and peri-synaptic space, preventing the synapse from becoming saturated and reducing the noise-to-signal ratio necessary to transmit information accurately. Therefore, clearance becomes essential for maintaining network stability and is important for the fidelity of computations performed by neuronal circuits [231].

Clearance of synaptic by-products from the brain produced by waking behavior is one of the clearest examples of how clearance relates to cognition. Ongoing neuronal activity will continually produce extracellular metabolites, reactive oxygen species, and misfolded proteins. These by-products, if cleared inefficiently, will negatively impact the ability of receptors to traffic properly, alter ion homeostasis, and affect the physical and chemical composition of the extracellular matrix [232,233]. Thus, the slow perivascular convective flow caused by vasomotion promoted by neuromodulator-dependent changes in blood vessel diameter will remove these by-products and maintain the local ionic environment required for proper synaptic transmission. However, failure to clear these by-products efficiently will lead to problems in network organization, leading to problems in synchronization, excitability, and signal-to-noise ratios that do not require significant structural damage and manifest clinically as initial difficulties in attention, working memory, and cognitive flexibility [234].

Clearance processes also interact with synaptic scaling and plasticity. The spatio-temporal distribution and rate of turnover of matrix metalloproteinases, neurotrophic factors, and extracellular vesicles carrying microRNAs and synaptic regulatory proteins are regulated by perivascular flow [235]. These regulators control the reformation of dendritic spines, the strength of long-term potentiation, and synaptic pruning. Therefore, the vasomotor clearance of waste products exerts effects on synaptic reorganization that are similar to the effects of synaptic activity itself. Therefore, alterations in perivascular flow can alter the threshold of plasticity and reduce learning efficiency, establishing a link between clearance kinetics and the time courses of learning and memory acquisition throughout the life span [236].

AQP4 dependent water transport and astrocyte derived calcium signals provide additional connections between clearance processes and neural activity. AQP4 polarization establishes a gradient in perivascular pressure that controls the fraction of the volume of the extracellular space available for the diffusion of neurotransmitters, the extent of ephaptic coupling, and the degree of cross talk between synapses [54]. Additionally, noradrenergic input to astrocytes regulates the geometry of the perivascular space, thereby regulating the hydraulic resistance and creating optimal conditions for synaptic signaling. Together, these results show that glia–perivascular interaction is an active player in the processing of cognitive information, rather than a mere passive supporter [130].

These results have recently been expanded to include oscillatory network dynamics, particularly those that relate to attention and memory. Oscillations in vasomotor pressure in the infra-slow range (0.01–0.1 Hz) are synchronized with infra-slow fluctuations in cortical excitability and with theta–gamma coupling in the hippocampus [237]. This indicates that the pressure waves caused by clearance may play a role in coordinating large scale populations of neurons through either the regulation of the ionic composition of the extracellular space or through the regulation of network gain mediated by astrocytes. Thus, disruptions in the synchronization of these processes could disrupt the coordination of different regions of the brain and the temporal relationships between the activity of neurons in different regions, which are necessary for working memory, cognitive control, and flexible behavior [238].

Furthermore, the relationship between clearance and the systems level stability of recurrent networks indicates that perturbations of clearance can add thermal “noise” to network computations, disrupting attractor basins, inducing imperfect transitions between cognitive states and altering the repertoire of possible network configurations [239]. The mechanisms described above may also be responsible for the early and subtle cognitive dysfunction seen in individuals who are pre-clinically neuro-degenerative, where mild impairments in high frequency clearance processes are sufficient to cause detectable cognitive dysfunction without significant structural change. Thus, the vasomotor pump represents a latent factor in cognitive plasticity that may influence learning, attention, and memory even in the absence of overt pathology [199].

### 7.2. Sleep Architecture, Memory Consolidation, and the Temporal Orchestration of Clearance

Given the relationship between clearance and sleep, the vasomotor pump is not only activated during sleep but is also integrated into the structural and temporal organization of sleep itself. The implications of this relationship are broad and, in addition to memory consolidation, clearance dynamics also influence synaptic reorganization and metabolic re-equilibration, all of which are fundamental to the maintenance of cognitive health and brain longevity [240].

During NREM sleep, the LC undergoes a transition from tonic firing to infra-slow oscillatory firing caused by intrinsic membrane currents and reciprocal inhibition between inhibitory cells. The LC oscillations are timed to the cortical down-states, thalamocortical spindles, and hippocampal sharp wave ripples, a process which forms a hierarchical temporal sequence that is suitable for memory replay and synaptic renormalization [241]. The vasomotor pump is part of this hierarchy. The adrenergic oscillations caused by the LC create slow vasomotion and the pressure gradients created by the vasomotion are at their greatest during cortical down-states when the population activity of neurons is at its lowest and when the energy requirements of the organism are at their lowest. Therefore, perivascular flow is enhanced at times when clearance is energetically advantageous and least disruptive to the ongoing synaptic processing [242].

This temporal relationship extends beyond the removal of metabolites. The perivascular flow influences the distribution of neuromodulators and extracellular vesicles, and therefore influences the timing and location of the signaling cascades involved in synaptic plasticity and memory consolidation [243]. In addition, clearance dynamically regulates the concentration of K^+^ and H^+^ in the extracellular space, both of which are strong regulators of the excitability of neurons and the oscillations of networks. Together, these processes indicate that clearance mechanisms are not only permissive for sleep-dependent plasticity, but are also active components in the cellular and network events that facilitate memory consolidation [244].

Optogenetic and pharmacological manipulations of LC firing during sleep have provided evidence for this perspective. Artificial restoration of the infra-slow noradrenergic oscillations causes an enhancement of perivascular flow and improved memory consolidation of spatial memories, whereas disruption of the LC oscillations impairs clearance and memory retention independent of the total duration of sleep [71]. These results establish a mechanistic link between clearance and the mnemonic functions of sleep and suggest that therapeutic manipulation of the vasomotor pump may enhance memory consolidation in the aged or diseased.

In addition to NREM sleep, clearance dynamics may also be involved in the synaptic remodeling that occurs during REM sleep. Although LC activity is low during REM sleep, the persistence of pressure oscillations caused by residual vascular compliance and respiratory coupling suggests that there is still some clearance occurring in this state [245]. Although the residual clearance may be insufficient to support widespread signaling and remodeling processes that occur during NREM sleep, it may be sufficient to support localized signaling and remodeling processes that differ in character from those occurring during NREM sleep. Therefore, clearance may be involved in all stages of the sleep cycle [6].

### 7.3. Neuroimmune Communication, Systemic Signaling, and the Expansion of the Clearance Paradigm

The noradrenergic vasomotor pump is not only involved in removing metabolic waste products. It is also deeply involved in neuroimmune communication, extracellular signaling, and systemic physiology. Pathophysiological disturbances in this system extend beyond traditional neurodegenerative disease to include a variety of psychiatric disorders, infections, and systemic inflammatory conditions [246].

Perivascular flow serves as a primary route for antigen drainage and immune surveillance. The flow transports solutes and immune mediators from the brain parenchyma to the meningeal and deep cervical lymphatics, thereby determining the degree of antigen presentation, adaptive immune priming, and communication between the central and peripheral immune compartments [247]. Slowed clearance of antigens will slow antigen trafficking, distort cytokine gradients, and alter the recruitment of immune cells, and may contribute to chronic neuroinflammation, impaired clearance of pathogens, and aberrant immune tolerance. These mechanisms help to explain the dysregulation of neuroimmune responses in a number of conditions, including multiple sclerosis, viral encephalitis, and post-infectious cognitive syndromes. Clearance also influences systemic physiology through the molecular cargo transported through clearance. Extracellular vesicles containing microRNAs, growth factors, and peptide signaling molecules move along perivascular flow and enter the peripheral circulation via the meningeal lymphatics, where they can modulate peripheral immune states, metabolic pathways, and possibly vascular tone. These findings indicate that the brain may influence the state of remote organs through clearance-dependent signaling [227,248]. In the CNS, clearance determines the spatial and temporal distributions of extracellular vesicles and signaling mediators involved in synaptic plasticity, neurogenesis, and glio-transmission. Therefore, altered clearance dynamics can affect the operation of particular signaling pathways and influence adult neurogenesis in the subventricular zone, microglial synaptic pruning, and long-range astrocyte–neuron communication [249]. These phenomena illustrate how clearance not only contributes to the maintenance of homeostatic constancy, but also to the adaptation and plasticity of behavior. There is now a growing literature which suggests that clearance dynamics may be involved in psychiatric pathology. There is evidence that disturbances in noradrenergic tone, decreased vascular compliance, and hyperpolarized AQP4 expression are present in major depression, schizophrenia, and bipolar disorder [250]. These disturbances in clearance would be expected to compromise trans-cellular and extra-cellular ionic homeostasis, disrupt synaptic signaling, and disrupt immune signaling cascades. These disruptions may make the networks more unstable and prone to abnormal patterns of activity. Therefore, modulating clearance dynamics may represent a new area of therapeutic intervention in psychiatric disorders, many of which are presently defined primarily in terms of neurotransmitter and receptor abnormalities [251].

The systemic implications of clearance clearly go beyond the CNS, and age-related declines in perivascular flow not only directly impair cerebral homeostasis, but also impair the trafficking of extracellular vesicles and cytokines involved in systemic inflammatory states (“inflammaging”), vascular stiffness, and immuno-senescence. Enhancing clearance may therefore have advantages that extend beyond the CNS, and potentially improve systemic metabolic health, immune competency, and vascular resilience [252].

Collectively, these findings indicate that a new paradigm may exist. The vasomotor pump can be viewed as a bidirectional signaling nexus between the brain and the body, and serves as a means of clearing solutes as well as a conduit for inter-organ communication. This connectivity may allow for coordinated regulation of neural, immune, and systemic physiological processes, and presents a fundamentally integrating model for understanding a wide range of diseases and conditions that are presently considered to be distinct neurological, psychiatric, and systemic diseases [253].

#### Conclusion of Section 7

The demonstration of the existence of a vasomotor pump, which is under noradrenergic control, serves to mark significances which extend far beyond any multiplicative factor offered by neurodegenerative pathology. It serves to show the existence of clearance as a phenomenon of the greatest importance, actively regulated in the state of brain. It is mediated by means of cognition, synaptic plasticity, memory assimilation, sleep architecture, immune inter-communication and systemic physiology. It relates perivascular flow in the brain as an instance of metabolic necessity on the one hand, and, on the other, a medium of signaling, thereby integrating neuronal activity with vascular dynamics, glial activities, immune function and inter-organ communication.

It is to be hoped that these new aspects of knowledge will serve to efface the borders between neurophysiology, immunology, and systems biology, thereby serving to show that a modulative approach in clearance functions may offer opportunities for influence in cognition, in the pathology of psychiatric disease processes, in systemic pathology of ageing, and in immune homeostasis. There will be mechanisms forthcoming by which the quantification and manipulations of the function of the pump will become realizable. There will be opportunities for influence, not only indicating the proper action of pathology in brain, but also pointing to possibilities of augmented influence in respect of brain function, the prolongation of cognitive longevity, and the envisagement of the functional role played by the brain in the body. If such opportunities are opened up to us, the vasomotor pump ceases to be a mere mechanism of clearance, but becomes an instrument of fundamental integrative character for neural health and functional performance and for systemic health.

## 8. Conclusions and Perspectives

The recognition of the vasomotor pump as an active neuro-modulatory rhythmically-driven process provides a revolutionary concept for brain clearance, a departure from considering clearance as a passive circulatory by-product. Rather than a passive by-product of circulation, clearance now becomes a physiologically regulated process shaped by rhythmic processes in the locus coeruleus, vascular biomechanical properties, astrocytic polarity, and perivascular compartment geometry. Therefore, a reduction in clearance is not merely coincidental but represents an early event in the continuum of aging and disease, thus providing a different model for understanding the maintenance of brain homeostasis, the adaptation of brain homeostasis to metabolic demand, and the deterioration of brain homeostasis throughout the lifespan.

Clearance is fundamentally embedded in the essential physiology of the brain. Clearance supports the ionic and metabolic environments necessary for stable synaptic transmission and neural network functioning. Clearance is involved in the formation of sleep patterns and memory consolidation. Clearance has a role in the surveillance of the immune system through the drainage of antigens, and influences communication between the CNS and systemic physiology. Thus, clearance cannot be considered simply as a background function, but rather as an active regulatory mechanism, which enables cognition, plasticity, and adaptive behaviors throughout the lifespan. The paradigm shift of understanding clearance as an actively regulated physiological function demonstrates common principles that connect previously distinct disciplines. Electrophysiology is connected to hemodynamics, glial biology to immunology, and sleep regulation to vascular mechanics. Early loss of clearance functions, potentially years before obvious pathology, may lead to the initial development of protein aggregation and degeneration of neurocytes. Therefore, the same mechanisms may underlie a wide variety of conditions, including Alzheimer’s and Parkinson’s disease, traumatic brain injury, and psychiatric disorders associated with poor noradrenergic drive, decreased vascular compliance, and disrupted astrocyte organization. There are multiple therapeutic targets available within the modular structure of the vasomotor pump. Pharmacological or targeted stimulation may restore lost neuro-modulatory rhythms. Decreased vascular compliance may be reversed by altering extracellular matrix composition or reprogramming the phenotype of smooth muscle cells. Astrocyte polarity and perivascular geometry may be improved through alteration of AQP4 trafficking and inflammatory pathways. Therefore, coordination of interventions addressing each domain may be capable of restoring clearance functions even in later stages of disease.

Due to recent technological advancements, these therapeutic opportunities have become increasingly feasible. High-resolution ultra-slow fMRI and phase contrast MRI allow for the non-invasive visualization of vasomotor oscillations and perivascular blood flow in living subjects. Noradrenergic integrity and astrocyte activity may be measured using biomarkers of noradrenergic tone, AQP4 localization, and exosome cargo. Gene editing, nanotechnology and bio-electronics provide the tools for precise modulation of the individual components of the pump. More importantly, the concepts presented here are not limited to disease. If perivascular dynamics regulate set-point scaling, network oscillation, and cognitive flexibility, then modulation of clearance may provide a way to enhance memory, attention and learning in normal individuals. Similarly, if clearance regulates antigen drainage and exosome signaling, then restoration of clearance may result in a re-setting of the tone of the immune system and increased resilience to systemic physiological challenges. Therefore, the concepts presented here suggest that clearance may be a therapeutic target for improving the health-span of cognitive and systemic function.

Finally, this paradigmatic shift also presents new ways to classify disease. Instead of classifying diseases solely based on the proteins they produce or their clinical manifestations, they may be classified based on the mode of failure of clearance—whether neuro-modulatory, vascular, glial or a combination. Therefore, a classification based on modes of clearance failure may unite clinically disparate conditions under common mechanistic frameworks, facilitating earlier diagnosis, more sensitive outcome measurements, and therapeutic interventions focused on the restoration of physiological function. However, despite these developments, there are still many unresolved questions. For example, how do neuro-modulatory rhythms, vascular mechanisms, and astrocyte polarity evolve together over the lifespan? At what threshold does reversible loss of clearance become irreversible loss? What is the contribution of the perivascular transport system to the priming of the immune response and to the communication between neuronal and extra-neuronal compartments? Finally, can specific enhancement of clearance significantly improve cognitive performance or slow the effects of aging in normal individuals? Answering these questions will require collaborative and interdisciplinary research at the intersection of molecular biology, physiology, biomedical engineering, and clinical science.

Furthermore, a broader scientific view requires a humble approach to understanding the vasomotor pump. The vasomotor pump is not a singular conduit but a complex system, whose stability arises from the coordinated interaction of multiple neuronal, vascular, glial and systemic elements. Therefore, therapeutic approaches must account for this complexity. Although we anticipate that improvements in our tools for measuring and controlling clearance will allow us to manipulate cognitive stability, neuro-immune interactions, and the effects of aging, the majority of the molecular landscape remains unexplored, and its complete physiological integration is just beginning to emerge.

Therefore, the purpose of this review is not a conclusion but an invitation—to continue investigating the mechanisms by which the brain maintains its internal environment, to identify the origin of clearance failure in disease, and to consider how this knowledge will ultimately shape the future of neuroscience. Undoubtedly, there remain undiscovered regulatory architectures in the most well-studied organ systems. The noradrenergic vasomotor pump is one such example—a dynamic interface connecting neuronal activity, vascular function, glial organization, and systemic physiology. The emergence of the vasomotor pump as a unifying principle encourages a re-evaluation of complex disorders and emphasizes the need for interdisciplinary approaches that span the cellular, network, and organismal levels of brain function.

## Figures and Tables

**Table 1:** Summary of the multiscale organization of the noradrenergic vasomotion axis.

Level/Component	Structural/Molecular Basis	Primary Clearance Function	Dominant Frequency	Dynamic Mechanism	Physiological Output	Pathological Consequence	References
Locus Coeruleus (LC)	Noradrenergic neurons with tonic + infralow firing (α_1_, β-AR projections)	Central oscillator coordinating vascular + glial rhythms	0.01–0.1 Hz	NE volume transmission entrains vascular and astrocytic activity	Synchronized vascular tone and perivascular conductance	LC degeneration, sleep loss, inflammation → loss of vasomotor drive	[51]
Vascular Smooth Muscle Cells (VSMCs)	α_1_-AR → Gq/PLC/IP_3_/Ca^2+^; β_2_-AR–cAMP modulation	Generates vasomotion (contraction–relaxation cycles)	0.01–0.1 Hz	Periodic Ca^2+^ cycling drives actomyosin contraction	Diameter oscillations generate convective pressure gradients	Stiffening → damped vasomotion, reduced hydraulic force	[52]
Pericytes	α_1_-AR, TRPV4; actin–myosin contractility	Fine-tuning of capillary resistance	0.01–0.05 Hz	Local contractility propagates arteriolar waves	Phase-coherent microvascular perfusion	Pericyte loss → heterogeneous capillary constriction, impaired clearance	[53]
Astrocytic End-feet	AQP4, α_1_/β-AR, Ca^2+^-dependent cytoskeletal remodeling	Controls perivascular permeability + geometry	0.03–0.1 Hz	NE-driven Ca^2+^ waves modulate AQP4 and end-foot volume	Adjusts perivascular resistance; CSF–ISF coupling	AQP4 depolarization → increased resistance, glymphatic failure	[54]
Perivascular Spaces (PVS)	Basement-membrane channels (10–30 μm)	Conduit for CSF influx and ISF mixing	Composite: cardiac (5–10 Hz), respiratory (0.2–0.3 Hz), vasomotor (0.01–0.1 Hz)	Integrated multi-frequency pressure waves sustain flow	Efficient solute transport periarterial → perivenous	Loss of compliance → low mixing, metabolite retention	[55]
Venular and Lymphatic Outflow	Perivenous pathways + meningeal lymphatics	Mediates ISF efflux and immune drainage	0.01–0.2 Hz	Passive venous oscillations + lymphatic pumping	Metabolite and antigen clearance	Venous rigidity/lymphatic obstruction → neuroinflammation	[56]
Network Integration	Coupled LC–vascular–glial feedback loops	Aligns neuro-modulatory, mechanical, hydrodynamic rhythms	Hierarchical (5–0.01 Hz)	Phase coupling across brain states	Sleep-dependent clearance optimization	Sleep fragmentation/aging → loss of coherence, clearance collapse	[57]

**Table 2:** Summary of key experimental evidence for noradrenergic vasomotion–driven brain clearance.

Approach	Key Findings	Main Limitations
Optogenetic LC stimulation	Infra-slow (~0.05 Hz) activation drives synchronized arteriole dilation and tracer influx, establishing a causal link between LC rhythm and perivascular flow.	Invasive cranial windows; rodent-only; anesthesia and stimulation may not fully reflect physiological LC activity.
Chemo-genetic (DREADD) manipulation	Excitatory DREADDs enhance vasomotion and solute movement; inhibitory DREADDs suppress both, confirming adrenergic control of clearance.	Alters LC excitability with prolonged use; slower temporal precision; possible off-target effects.
α_1_-adrenergic pharmacology	α_1_-agonists restore vasomotion after LC suppression; α_1_-blockers abolish oscillations despite intact LC firing, identifying α_1_ receptors as essential effectors.	Limited receptor selectivity; systemic cardiovascular effects; pharmacologic tone may differ from physiological conditions.
Two-photon microscopy	Directly visualizes diameter oscillations and tracer motion at 0.01–0.1 Hz, demonstrating spatiotemporal coupling of mechanics and solute transport.	Highly invasive; restricted field of view; anesthesia alters noradrenergic tone and vessel compliance.
Dynamic contrast/phase-contrast MRI	Reveals whole-brain slow CSF–ISF oscillations peaking during NREM sleep, supporting large-scale vasomotor synchronization.	Flow measurements are indirect; limited temporal resolution; cardiac and respiratory confounds.
Biophysical and mechanical modeling	Accurate tracer kinetics reproduced only when slow vasomotion is included, supporting mechanical sufficiency of LC-driven pressure waves.	Simplified assumptions; lacks biological feedback; species-specific parameter variability.
Transgenic disease models	LC loss or reduced vasomotion precedes impaired clearance; restoring LC rhythmicity delays pathology and improves recovery.	Species differences; anesthesia and genetic background affect progression; short lifespan restricts long-term study.
Human neuroimaging	Detects infra-slow vasomotion linked to sleep; LC signal declines with aging and Alzheimer’s disease, paralleling reduced clearance.	Correlational; insufficient resolution to define cellular mechanisms or establish causality.

**Table 3:** Disintegration at hierarchical levels and partial reversibility of the noradrenergic vasomotor pump in aging and disease.

Domain/Component	Primary Pathological Process	Molecular and Cellular Mechanisms	Functional Consequence	Quantitative/Experimental Evidence	Potential Reversibility/Therapeutic Target	References
Locus Coeruleus (LC)	Degeneration of noradrenergic neurons and rhythm desynchronization	Mitochondrial dysfunction, oxidative stress, impaired mitophagy, microglial activation, reduced HCN/T-type Ca^2+^ currents	Loss of infra-slow oscillations (0.01–0.1 Hz); weakened NE drive	LC neuron loss: >40% by 7th decade; reduced NE tone precedes AD pathology by 10–20 years	Chemo-genetic activation or NE reuptake inhibitors restore vasomotor coupling	[51,146]
Adrenergic Signaling Cascade	Receptor desensitization and impaired α1/β2 balance	Downregulation of α1-AR on VSMCs and β2-AR on astrocytes; G-protein uncoupling	Reduced vasomotor amplitude and phase coherence	In aged mice, vasomotor amplitude ↓ ~70%; α1-AR mRNA ↓ ~60%	α1 agonists, β2 modulators, LC–astrocyte co-stimulation	[147]
Vascular Smooth Muscle Cells (VSMCs)	Phenotypic shift and cytoskeletal degeneration	Contractile-to-synthetic transition; actin loss; Ca^2+^ handling defects; ROS accumulation	Damped vasomotion; decreased compliance; transmission failure	Penetrating arteriole oscillations ↓ to <30% of young controls	NO donors, ROCK inhibitors, TGF-β blockade	[148]
Vascular Matrix and Elasticity	Elastin fragmentation, collagen crosslinking, calcification	AGE accumulation, MMP activation, angiotensin II signaling	Elevated stiffness, loss of low-frequency resonance	Arterial compliance ↓ 40–60% with age; loss of 0.01–0.1 Hz response	Crosslink breakers (ALT-711), sirtuin activators	[149]
Pericytes/Capillary Network	Loss of contractility and dropout	TRPV4 desensitization, oxidative DNA damage, apoptosis	Impaired microvascular resistance tuning; flow heterogeneity	Pericyte density ↓ ~25–40% in aging cortex	PDGF-BB therapy, pericyte reprogramming	[150]
Astrocytic End-feet and AQP4 Polarity	Depolarization and cytoskeletal rigidity	Disruption of dystrophin–dystroglycan complex; IL-1β/TNF-α–driven astrogliosis	Increased hydraulic resistance, reduced ISF–CSF exchange	AQP4 mislocalization ↑ 200–300% in AD cortex; tracer clearance ↓ > 50%	Dystrophin restoration, AQP4 modulators, astrocyte reprogramming	[131,151]
Perivascular Space Geometry	Fibrosis and basement membrane thickening	Collagen IV accumulation, perivascular inflammation, MMP imbalance	Narrowed lumen, elevated impedance, stagnation zones	PVS cross-section ↓ ~35% in aged rodents; diffusion times ↑ 2–3×	Anti-fibrotic, anti-MMP, or lymphatic drainage enhancers	[152]
Network-Level Integration	Phase decoupling and loss of multiscale synchrony	Desynchronization between LC rhythms, vascular oscillations, astrocytic Ca^2+^ waves	Transition from convective to diffusion-limited clearance	Net CSF flow velocity ↓ > 60% in aged animals; clearance half-time ↑ 3×	Combined neuro-modulatory–vascular–astro-glial restoration	[114]

## Data Availability

No new data were created or analyzed in this study. Data sharing is not applicable to this article.
